# The Stable Level of *Glutamine synthetase 2* Plays an Important Role in Rice Growth and in Carbon-Nitrogen Metabolic Balance

**DOI:** 10.3390/ijms160612713

**Published:** 2015-06-04

**Authors:** Aili Bao, Zhuqing Zhao, Guangda Ding, Lei Shi, Fangsen Xu, Hongmei Cai

**Affiliations:** 1 Microelement Research Center, Key Laboratory of Arable Land Conservation (Middle and Lower Reaches of Yangtze River), Ministry of Agriculture, Huazhong Agricultural University, Wuhan 430070, China; E-Mails: baoaili19870212@163.com (A.B.); zzq@mail.hzau.edu.cn (Z.Z.); dgd@mail.hzau.edu.cn (G.D.); Leish@mail.hzau.edu.cn (L.S.); fangsenxu@mail.hzau.edu.cn (F.X.); 2 National Key Laboratory of Crop Genetic Improvement, Huazhong Agricultural University, Wuhan 430070, China

**Keywords:** carbon-nitrogen, chlorosis, gene expression, *GS2*, metabolic profile, rice

## Abstract

Glutamine synthetase 2 (GS2) is a key enzyme involved in the ammonium metabolism in plant leaves. In our previous study, we obtained *GS2*-cosuppressed plants, which displayed a normal growth phenotype at the seedling stage, while at the tillering stage they showed a chlorosis phenotype. In this study, to investigate the chlorosis mechanism, we systematically analyzed the plant growth, carbon-nitrogen metabolism and gene expressions between the *GS2*-cosuppressed rice and wild-type plants. The results revealed that the *GS2*-cosuppressed plants exhibited a poor plant growth phenotype and a poor nitrogen transport ability, which led to nitrogen accumulation and a decline in the carbon/nitrogen ratio in the stems. Interestingly, there was a higher concentration of soluble proteins and a lower concentration of carbohydrates in the *GS2*-cosuppressed plants at the seedling stage, while a contrasting result was displayed at the tillering stage. The analysis of the metabolic profile showed a significant increase of sugars and organic acids. Additionally, gene expression patterns were different in root and leaf of *GS2*-cosuppressed plants between the seedling and tillering stage. These results indicated the important role of a stable level of *GS2* transcription during normal rice development and the importance of the carbon-nitrogen metabolic balance in rice growth.

## 1. Introduction

Nitrogen (N) is one of the essential macronutrients required for plant growth. It is also a major limiting factor, which determines plant biomass and crop yield [1,2]. N is not only a constituent of key molecules, such as amino acids, nucleic acids, chlorophyll, ATP and several plant hormones, it is also the regulator in many biological processes, such as amino acid metabolism, carbon metabolism and protein synthesis [3,4]. Additionally, carbon (C) plays a crucial role in plants’ routine growth and development. Various C compounds, including glucose, sucrose, organic acids and other carbohydrates, provide both the C skeletons and the energy for ammonium (NH_4_^+^) assimilation. N compounds are synthesized by incorporating NH_4_^+^ into the C skeletons. Amino acids and proteins, in particular enzymes, are essential for almost all cellular activities, including the C and N metabolic reactions [5]. Therefore, both C and N nutrients are essential for cellular functions. Recently, it has been recognized that cellular C and N metabolism must be tightly coordinated. Maintaining a coordination of carbon-nitrogen metabolism and an appropriate balance of carbohydrates to nitrogen metabolites, which also is referred to as the “C/N balance”, is important for plant growth, development and yield production [5,6,7,8,9,10].

Glutamine synthetase (GS; EC 6.3.1.2) is a key enzyme for the assimilation of NH_4_^+^, which is derived from nitrate (NO_3_^−^), nitrogen fixation or generated by other reactions that release ammonium within the plant, into glutamine (Gln) [11,12,13]. The GS/GOGAT (glutamate synthase) cycle can generate two molecules of glutamate (Glu). One, as the substrate, is cycled back to the NH_4_^+^ assimilation by the GS enzyme; the other one is exported out of the GS/GOGAT cycle to synthesize other types of amino acids [14]. In higher plants, there are two GS isoforms, cytosolic GS1 and chloroplastic GS2 [15,16]. Generally, there is a multigene family that encodes the cytosolic GS1, while only a single gene encodes the chloroplastic GS2 [17,18,19,20,21,22,23]. There is one exception that *Medicago truncatula* has two *GS2* genes [24]. To date, only the *OsGS2* gene is identified in rice that encodes chloroplastic GS2, which is mainly expressed in leaf [25].

Several studies have justified the importance of the GS2 enzyme in the assimilation of ammonium, especially the major function in photorespiratory ammonium re-assimilation, because mutants lacking the *GS2* gene were able to grow normally under non-photorespiratory conditions. For example, high light intensity tolerance and photorespiration capacity were observed in *GS2*-overexpressed transgenic tobacco plants [26]. Similarly, increased salt tolerance and photorespiration capacity were also found in transgenic rice [27]. The amount of GS2 protein decreased by 35% in *GS2*-silencing oil seed rape plants, while no changes in plant growth were observed when compared to the wild-type plants [28]. *GS2* deficiency in *Lotus japonicas* has an important effect on the nodulation process in legumes [29]. Moreover, barley *GS2* mutants died when grown in normal air, because they were unable to re-assimilate photorespiratory ammonium [30,31,32]. A recent study indicated that *GS2* genes may be related to the grain protein content of durum wheat [23].

Plant growth and crop production requires abundant nitrogen, which is generally the most common limiting nutrient for the growth and yield of crops worldwide. Large amounts of nitrogen fertilizers are applied to meet the high nitrogen requirements of crop plants. However, the application of large quantities of fertilizers to increase crop yield is not economically sustainable and also leads to environmental pollution. Reports pointed out that less than half of the applied nitrogen fertilizers were absorbed and used by crop plants, and the left nitrogen was inevitably lost to the atmosphere and leached into the underground water system, leading to severe environmental pollution [33]. A recent analysis showed that high N fertilizer input was the main reason for soil acidification in China [34]. Rice (*Oryza sativa*) is the main staple food in the world. As rice has the smallest genome among the major cereals and amount of genetic resources, it becomes a model cereal for science research [35]. Because of the important function of the GS enzyme in plant N metabolism, particular attention has been devoted to studies on GS transformation in higher plants, which is expected to be a good molecular method to analyze gene functions and a good strategy to improve nitrogen use efficiency.

Based on these aspects, we constructed the *GS2*-overexpressed transformants using *CaMV*35S promoter and obtained the transgenic rice plants by the *Agrobacterium*-mediated transformation method to improve the nitrogen use efficiency [36]. Interesting results were observed in these transgenic plants. In the T_0_ generation, transcripts of the *GS2* gene were shown to accumulate at higher levels, and no differences in the visible growth phenotype were observed; whereas, in the T_1_ generation, the transgenic plants exhibited a chlorosis phenotype (yellow leaves) accompanied by a significant decline in the level of *GS2* messenger RNA (mRNA) and total GS activity, decreased plant height, few tillers and decreased dry weight. In addition, the transgenic plants displayed better performance when grown in a normal nutrient solution complemented with exogenous glutamine (Gln) [36]. Based on these results, we renamed the transgenic plants *GS2*-cosuppressed rice. In the present study, to determine the possible reason for the chlorosis phenotype of the transgenic plants, we systematically analyzed the differences in the plant growth, carbon-nitrogen metabolism and gene expression profile between the *GS2*-cosuppressed rice (homozygous T_2_ generation of transgenic Line 87 with a decrease of 28% in GS activity in leaves) and wild-type Zhonghua 11 at the seedling stage and tillering stage. Additionally, in order to test the different response of the transgenic plants to the inorganic and organic nitrogen supplied in the environment, we planted these plants hydroponically under different nitrogen forms (supplied with ammonium nitrate, glutamine and both nitrogen forms). The results indicated the important role of a stable level of transcription of *GS2* mRNA for the normal development of rice and the carbon-nitrogen metabolic balance. The unbalanced carbon-nitrogen metabolic status and the excessively high expression level of *GS2* mRNA in the root may be the reasons for the chlorosis phenotype in the *GS2*-cosuppressed plants.

## 2. Results

### 2.1. Growth Phenotype of GS2-Cosuppressed Plants at the Seedling and Tillering Stages

In our previous study, the *GS2*-cosuppressed plants exhibited a normal growth phenotype with a higher *GS2* mRNA transcriptional level at the seedling stage, while the chlorosis phenotype and a lower *GS2* mRNA transcriptional level was observed at the tillering stage [36]. Additionally, the *GS2*-cosuppressed plants displayed better performance when grown in a normal nutrient solution complemented with exogenous Gln [36]. To describe these phenotypes in detail, we measured the root length, plant height and the root and shoot dry weight of *GS2*-cosuppressed plants (homozygous T_2_ generation of transgenic Line 87 with a decrease of 28% in GS activity in leaves) and wild-type plants at the seedling stage and the tillering stage under N, G (Gln) and N + G conditions. Additionally, the leaf SPAD (Soil and Plant Analyzer Development) value and photosynthesis parameters were also examined in *GS2*-cosuppressed plants and wild-type plants at the tillering stage under N, G and N + G conditions.

**Figure 1:**
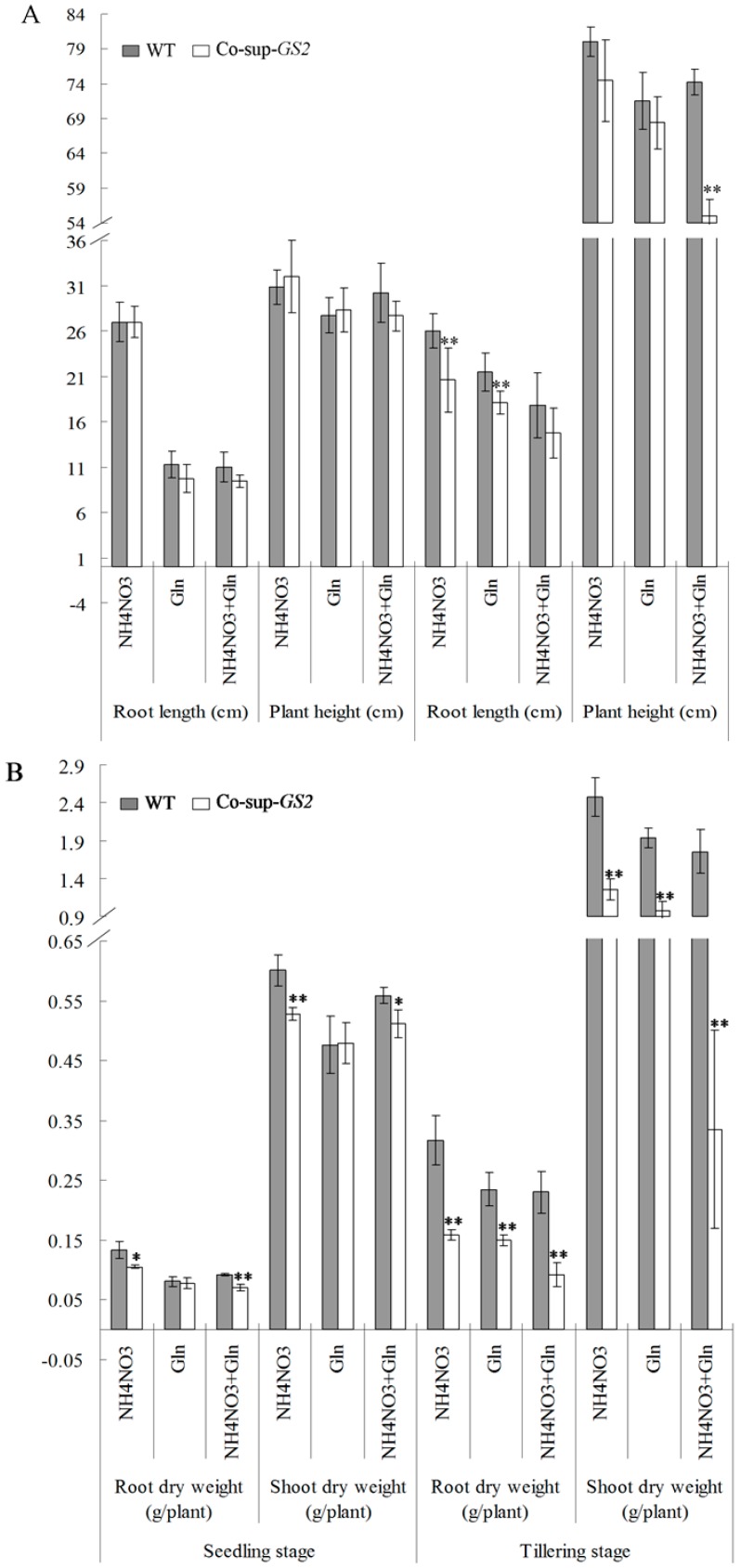
The root length and plant height (**A**), root dry weight and shoot dry weight (**B**) in the *GS2*-cosuppressed plants (Co-sup-*GS2*) and wild-type plants (WT) at the seedling stage and the tillering stage under N (NH_4_NO_3_), G (Gln) and N + G (NH_4_NO_3_ + Gln) conditions. Values are the mean ± SD of ten randomly-selected plants. *, ** Significant differences at the level of *p* = 0.05 and *p* = 0.01, respectively.

At the seedling stage, there was no obvious difference in the root length and plant height between the *GS2*-cosuppressed plants and wild-type plants (Figure 1A). At the tillering stage, there were significant (*p* < 0.01) decreases in the root length and plant height in *GS2*-cosuppressed plants compared to the wild-type plants (20.8% and 15.4% decreases in root length in the *GS2*-cosuppressed plants grown under N and G conditions, respectively; 25.9% decreases in plant height in *GS2*-cosuppressed plants grown under N + G conditions; Figure 1A). In addition, the root length of both *GS2*-cosuppressed plants and the wild-type plants decreased significantly after Gln was supplied to the nutrient solution, especially at the seedling stage. There were 58.3%–65% decreases in root length growth under G and N + G conditions when compared to the N condition (Figure 1A). For the root and shoot dry weight analysis, there were significant (*p* < 0.05) reductions in *GS2*-cosuppressed plants at both the seedling and tillering stages when compared to the wild-type plants under these three different growth conditions, except the root and shoot dry weight under the G condition at the seedling stage (Figure 1B). Compared to the wild-type plants, there were 20.8%–23.2% reductions in the root dry weight, and 8.6%–12.2% reductions in the shoot dry weight of *GS2*-cosuppressed plants were observed at the seedling stage; meanwhile, 36.2%–59.8% reductions in the root dry weight and 49.1%–80.9% reductions in the shoot dry weight of *GS2*-cosuppressed plants were observed at the tillering stage (Figure 1B). These results showed the different growth phenotypes between the *GS2*-cosuppressed plants and the wild-type plants at the tillering stage, especially with regard to the root and shoot dry weight.

The leaf SPAD value of the wild-type plants was stable under three different growth conditions, while the leaf SPAD value of the *GS2*-cosuppressed plants increased after the addition of Gln to the nutrient solution (Table 1). Additionally, the leaf SPAD value of the *GS2*-cosuppressed plants decreased significantly (*p* < 0.05) when compared to the wild-type plants, with 16.9%, 10.7% and 4.9% decreases for the N, G and N + G conditions, respectively (Table 1). However, there were no changes in the photosynthetic parameters (including photosynthetic rate, stomatal conductance, intercellular CO_2_ concentration and transpiration rate) between the *GS2*-cosuppressed plants and the wild-type plants, except there were significant (*p* < 0.01) decreases in the photosynthetic rate (14.6% decrease) and transpiration rate (20.6% decrease) in the *GS2*-cosuppressed plants grown under the G condition when compared to the wild-type plants (Table 1).

**Table 1:** The leaf SPAD value and photosynthetic parameters in the *GS2*-cosuppressed plants (Co-sup-*GS2*) and wild-type plants (WT) at the tillering stage under N (NH_4_NO_3_), G (Gln) and N + G (NH_4_NO_3_ + Gln) conditions.

Treatment	SPAD at Tillering Stage	Photosynthesis Parameters at Tillering Stage			
		Photosynthetic Rate (μmol CO_2_ m^−2^·s^−2^)	Stomatal Conductance (mmol m^−2^·s^−1^)	Intercellular CO_2_ Concentration (μL·L^−1^)	Transpiration Rate (mmol H_2_O m^−2^·S^−1^)
**NH_4_NO_3_**					
WT	46.1 ± 2.9	28.24 ± 3.32	0.82 ± 0.06	287.75 ± 9.62	15.06 ± 1.10
Co-sup-*GS2*	38.3 ± 6.3 **	29.76 ± 3.24	1.01 ± 0.26	292.91 ± 8.36	15.99 ± 1.36
**Gln**					
WT	46.0 ± 2.2	33.24 ± 2.10	0.90 ± 0.12	278.39 ± 7.79	16.36 ± 1.56
Co-sup-*GS2*	41.1 ± 3.2 **	28.39 ± 1.54 **	0.71 ± 0.05	277.77 ± 1.32	12.99 ± 0.61 **
**NH_4_NO_3_ + Gln**					
WT	44.5 ± 2.4	30.83 ± 2.03	0.76 ± 0.10	274.75 ± 9.98	14.70 ± 1.61
Co-sup-*GS2*	42.3 ± 2.0 *	29.53 ± 2.36	0.64 ± 0.02	268.03 ± 5.93	13.32 ± 0.74

Values are mean ± SD from ten randomly-selected plants. *, ** Significant differences at the level of *p* = 0.05 and *p* = 0.01, respectively.

### 2.2. Nitrogen Uptake by GS2-Cosuppressed Plants

**Figure 2:**
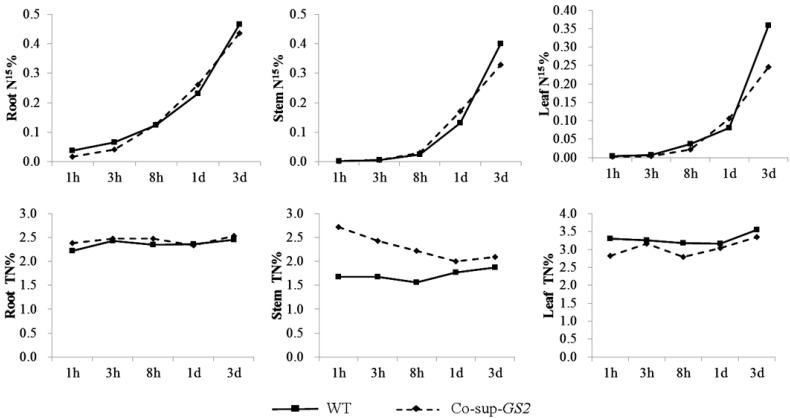
The ^15^N (^15^N%) and total nitrogen content (TN%) in the roots, stems and uppermost leaves of the *GS2*-cosuppressed plants (Co-sup-*GS2*) and wild-type plants (WT) at 1 h, 3 h, 8 h, 1 day and 3 days after NH_4_NO_3_ in the nutrient solution was replaced with ^15^NH_4_^15^NO_3_ during the tillering stage. The values are the means from three biological replicated plant materials.

As GS is the main enzyme that assimilates NH_4_^+^ into Gln, we analyzed the nitrogen uptake and transport ability of the *GS2*-cosuppressed plants at the tillering stage using ^15^N. The total carbon and nitrogen concentration and the carbon/nitrogen ratio were also determined in the roots, stems and leaves of *GS2*-cosuppressed plants and wild-type plants under N, G and N + G growth conditions at the tillering stage. For the nitrogen uptake assay, the NH_4_NO_3_ in the nutrient solution was replaced by ^15^NH_4_^15^NO_3_. After 1 h, 3 h, 8 h, 1 day and 3 days, the concentrations of total nitrogen and ^15^N in the roots, stems and leaves were analyzed in the *GS2*-cosuppressed plants and wild-type plants. The results showed that there was no difference in the total nitrogen concentration in the root between the *GS2*-cosuppressed plants and the wild-type plants. The stem total nitrogen concentration of the *GS2*-cosuppressed plants was 16.5%–61.9% higher than that of the wild-type plants; while the leaf total nitrogen concentration was 5.5%–16.6% lower than that of the wild-type plants (Figure 2). Similarly, there was no difference in the ^15^N concentration in the roots between the *GS2*-cosuppressed plants and the wild-type plants. The ^15^N concentration in the stems of the *GS2*-cosuppressed plants was 11.4%–66.7% higher than that of the wild-type plants; while 3 d after the NH_4_NO_3_ in the nutrient solution was replaced with ^15^NH_4_^15^NO_3_, the ^15^N concentration in the leaves of the *GS2*-cosuppressed plants was 25.0% lower than in the wild-type plants (Figure 2). For the total carbon and nitrogen concentration analysis, significant (*p* < 0.01) increases in the total nitrogen concentration (26.0%, 32.7% and 18.1%) and significant (*p* < 0.05) decreases in the carbon/nitrogen ratio (20.2%, 24.0% and 17.9%) in the stem were observed in the *GS2*-cosuppressed plants grown under N, G and N + G conditions, respectively (Table 2). In addition, 3.8%, 5.3% and 9.9% decreases in the total nitrogen concentration were also observed in the *GS2*-cosuppressed plant leaves grown under the N, G and N + G conditions, respectively (Table 2). These results indicated a similar nitrogen uptake ability in roots between the *GS2*-cosuppressed plants and the wild-type plants, while a poor nitrogen transport ability from stems to leaves was observed, which led to nitrogen accumulation (especially the NO_3_^−^; data not shown here) and a decrease in the carbon/nitrogen ratio in the stems of the *GS2*-cosuppressed plants.

**Table 2:** The carbon content (C%), nitrogen content (N%) and carbon/nitrogen ratio (C/N) in the roots, stems and uppermost leaves of the *GS2*-cosuppressed plants (Co-sup-*GS2*) and wild-type plants (WT) at the tillering stage under N (NH_4_NO_3_), G (Gln) and N + G (NH_4_NO_3_ + Gln) conditions.

Treatment	C%			N%			C/N		
	Root	Stem	Leaf	Root	Stem	Leaf	Root	Stem	Leaf
**NH_4_NO_3_**									
WT	38.64 ± 0.23	35.02 ± 0.52	38.81 ± 0.39	2.81 ± 0.13	2.50 ± 0.09	3.19 ± 0.05	13.75 ± 0.59	14.00 ± 0.41	12.16 ± 0.10
Co-sup-*GS2*	38.49 ± 0.49	35.11 ± 0.47	38.97 ± 0.80	3.16 ± 0.10	3.15 ± 0.15 **	3.07 ± 0.17	12.18 ± 0.53	11.17 ± 0.69 **	12.69 ± 0.41
**Gln**									
WT	41.18 ± 0.16	36.59 ± 0.30	39.78 ± 0.35	3.29 ± 0.20	2.84 ± 0.22	3.41 ± 0.10	12.53 ± 0.79	12.92 ± 0.86	11.68 ± 0.24
Co-sup-*GS2*	42.41 ± 0.30	37.00 ± 0.66	40.31 ± 0.73	3.21 ± 0.07	3.77 ± 0.07 **	3.23 ± 0.10	13.20 ± 0.40	9.82 ± 0.10 *	12.48 ± 0.23
**NH_4_NO_3_ + Gln**									
WT	41.92 ± ND	38.67 ± 0.36	42.64 ± 0.26	3.86 ± ND	3.70 ± 0.07	4.16 ± 0.06	10.86 ± ND	10.45 ± 0.17	10.25 ± 0.08
Co-sup-*GS2*	ND	37.54 ± 0.57	40.67 ± 1.14	ND	4.37 ± 0.13 **	3.75 ± 0.17	ND	8.58 ± 0.13 **	10.84 ± 0.19

Values are the mean ± SD from three biological replicated plant materials. *, ** Significant differences at the level of *p* = 0.05 and *p* = 0.01, respectively. ND: no data.

### 2.3. Soluble Proteins and Carbohydrates Determination in the GS2-Cosuppressed Plants

**Figure 3:**
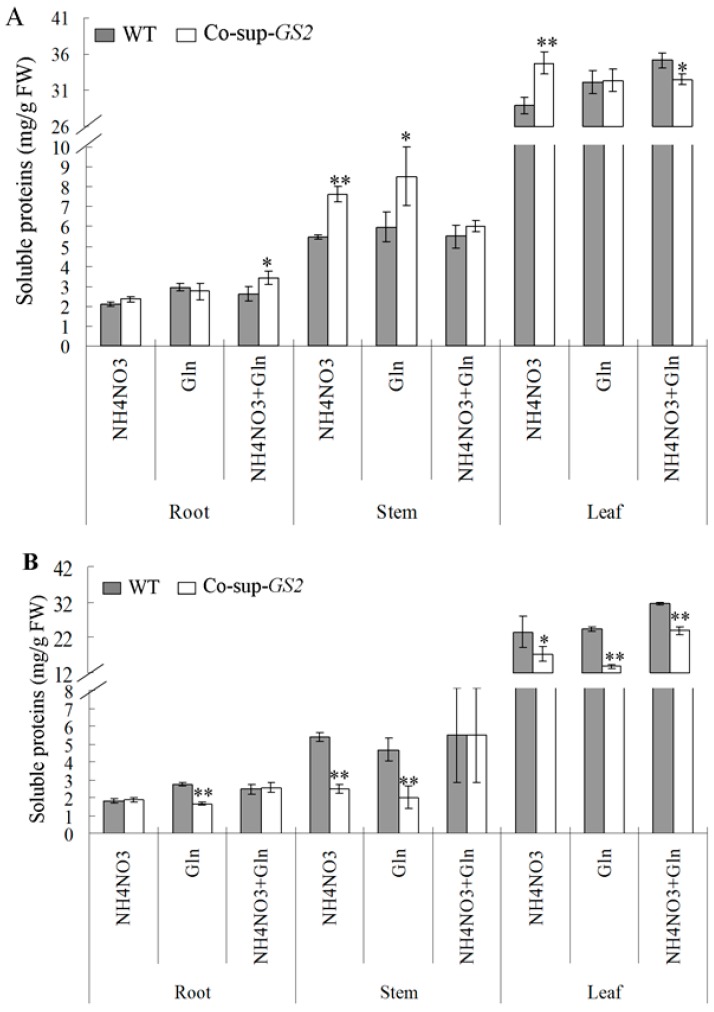
The concentration of soluble proteins in the roots, stems and uppermost leaves of *GS2*-cosuppressed plants (Co-sup-*GS2*) and wild-type plants (WT) at the seedling stage (**A**) and the tillering stage (**B**) under N (NH_4_NO_3_), G (Gln) and N + G (NH_4_NO_3_ + Gln) conditions. Values are the mean ± SD from three biological replicated plant materials. *, ** Significant differences at the level of *p* = 0.05 and *p* = 0.01, respectively.

To evaluate the differences in the carbon and nitrogen metabolic status between the *GS2*-cosuppressed plants and the wild-type plants, we determined the concentrations of total soluble proteins and soluble carbohydrates in the roots, stems and leaves of *GS2*-cosuppressed plants and wild-type plants under N, G and N + G growth conditions at the seedling and tillering stages. The results showed that there was a higher concentration of soluble proteins in the *GS2*-cosuppressed plants at the seedling stage, while there was a lower concentration of soluble proteins in the *GS2*-cosuppressed plants at the tillering stage compared to the wild-type plants (Figure 3). For example, at the seedling stage, compared to the wild-type plants, there was a 30.5% increase in the root soluble proteins in the *GS2*-cosuppressed plants under N + G conditions; there were 39.0% and 42.4% increases in soluble proteins in the stems under N and G conditions, respectively; and there was a 20.2% increase in the soluble proteins in the leaves under the N condition (Figure 3A). At the tillering stage, compared to the wild-type plants, there was a 39.8% decrease in the root soluble proteins of the *GS2*-cosuppressed plants under the G condition; there were 53.6% and 56.9% decreases in the soluble proteins in the stems under the N and G conditions, respectively; and there were 26.4%, 43.0% and 24.1% decreases in the soluble proteins in the leaves under the N, G and N + G conditions, respectively (Figure 3B).

**Figure 4:**
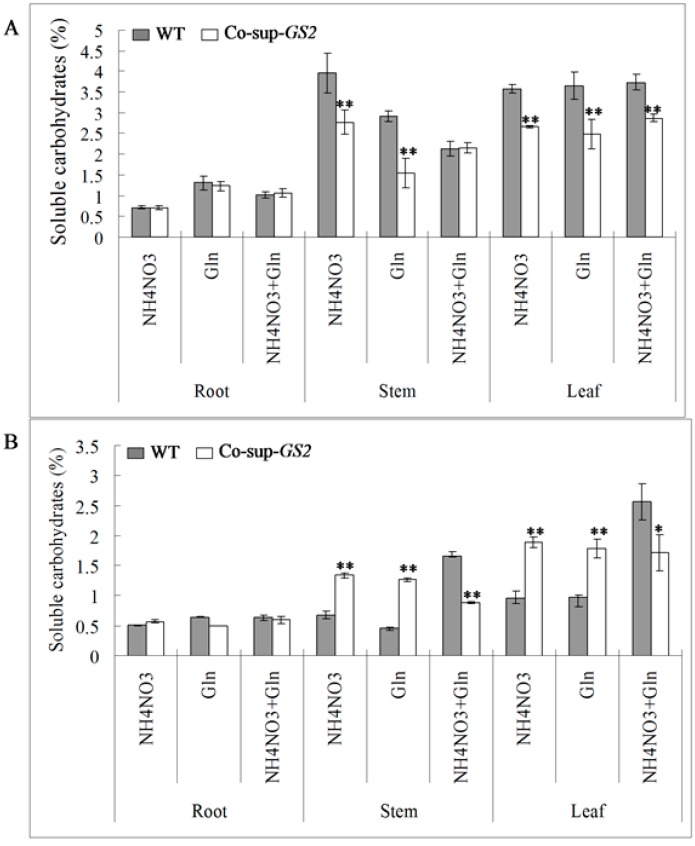
The concentration of soluble carbohydrates in the roots, stems and uppermost leaves of *GS2*-cosuppressed plants (Co-sup-*GS2*) and wild-type plants (WT) at the seedling stage (**A**) and the tillering stage (**B**) under N (NH_4_NO_3_), G (Gln) and N + G (NH_4_NO_3_ + Gln) conditions. Values are the mean ± SD from three biological replicated plant materials. *, ** Significant differences at the level of *p* = 0.05 and *p* = 0.01, respectively.

In contrast, there was a lower concentration of soluble carbohydrates in the *GS2*-cosuppressed plants at the seedling stage, while there was a higher concentration of soluble carbohydrates in the *GS2*-cosuppressed plants at the tillering stage when compared to the wild-type plants (Figure 4). For example, at the seedling stage, compared to the wild-type plants, there were 30.0% and 46.7% decreases in the stem soluble carbohydrates of *GS2*-cosuppressed plants under the N and G conditions, respectively; and there were 25.7%, 31.8% and 23.2% decreases in soluble carbohydrates in the leaves under the N, G and N + G conditions, respectively; (Figure 4A). At the tillering stage, compared to the wild-type plants, there were 99.9% and 178.0% increases in the soluble carbohydrates in the stem, and there were 98.3% and 84.6% increases in the soluble carbohydrates in the leaves of *GS2*-cosuppressed plants under the N and G conditions, respectively (Figure 4B). These results suggested that the altered *GS2* expression level (higher *GS2* expression level at the seedling stage and lower *GS2* expression level at the tillering stage) affected the carbon and nitrogen metabolic status. There was an imbalance between the soluble protein and carbohydrate concentrations in the *GS2*-cosuppressed plants, especially in the stems and leaves.

### 2.4. Metabolite Profiling Analysis in GS2-Cosuppressed Plants

**Figure 5:**
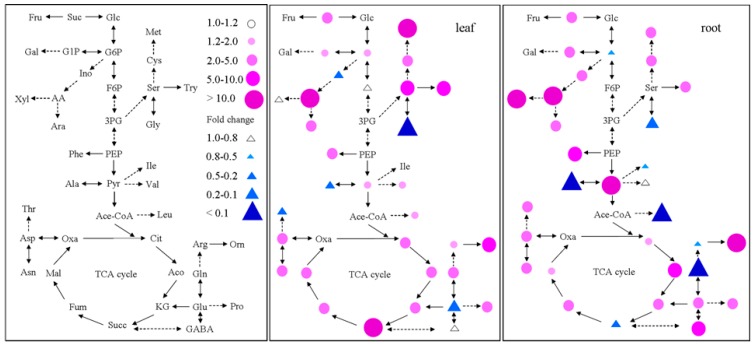
The fold change corresponds to the ratio of the concentration of individual metabolites involved in carbon and nitrogen metabolism in the *GS2*-cosuppressed plants relative to the wild-type plants for the uppermost leaves and roots at the tillering stage under N condition. Red dots indicate increased metabolites, and blue triangles indicate decreased metabolites. Glc, glucose; Suc, sucrose; Fru, fructose; F6P, frutose-6-P; G6P, glucose-6-P; G1P, glucose-1-P; Gal, galactose; Ino, inositol; AA, ascorbic acid; Ara, arabinose; Xyl, xylitol; 3PG, 3-P-glycerate; PEP, phosphoenolpyruvate; Pyr, pyruvate; Ace-CoA, acetyl-CoA; Cit, citrate; Aco, aconitase; KG, ketoglutarate; Succ, succinate; Fum, fumarate; Mal, malate; Oxa, oxaloacetate; Glu, glutamate; Gln, glutamine; Arg, arginine; Pro, proline; Orn, ornithine; GABA, aminobutyric; Asp, aspartate; Asn, asparagine; Ile, isoleucine; Met, methionine; Thr, threonine; Ala, alanine; Val, valine; Leu, leucine; Phe, phenylalanine; Try, tryptophan; Ser, serine; Gly, glycine; Cys, cysteine.

To study the individual metabolites involved in the carbon and nitrogen metabolic pathway in detail, we analyzed the sugars, organic acids and free amino acids in the root and leaf tissues of *GS2*-cosuppressed plants and wild-type plants at the tillering stage under different N conditions. Figure 5 and Supplementary Figure S1 display the fold change corresponding to the ratio of *GS2*-cosuppressed plants/wild-type plants, calculated using the concentrations of these individual metabolites. Dramatic increases in the sugars, organic acids and free amino acids were observed in both the leaf and root tissues of the *GS2*-cosuppressed plants compared to the wild-type plants, especially for ascorbic acid (>37.7-fold), succinate (>312.4-fold) and methionine (>410.3-fold) in the leaf and xylitol (>46.3-fold), ascorbic acid (>14.2-fold), pyruvate (>808.1-fold) and ornithine (>19.9-fold) in the root (Figure 5; Supplementary Figure S1). Meanwhile, several metabolites had dramatically decreased concentrations, including glutaric acid (<0.10-fold) and glycine (<0.07-fold) in the leaf and glutamine (<0.04-fold), alanine (<0.004-fold) and leucine (<0.0006-fold) in the root (Figure 5; Supplementary Figure S1). However, the concentrations of total sugars, total organic acids and total free amino acids increased only slightly, a fold change ranging from 2.0–3.7 (data not shown here).

### 2.5. Gene Expression Analysis in GS2-Cosuppressed Plants

**Figure 6:**
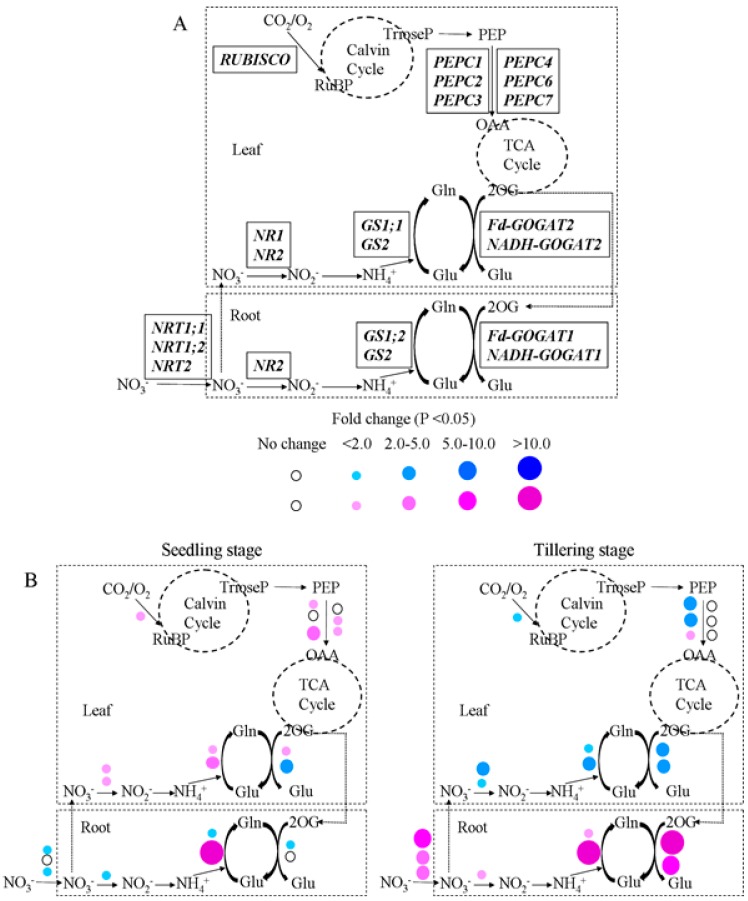

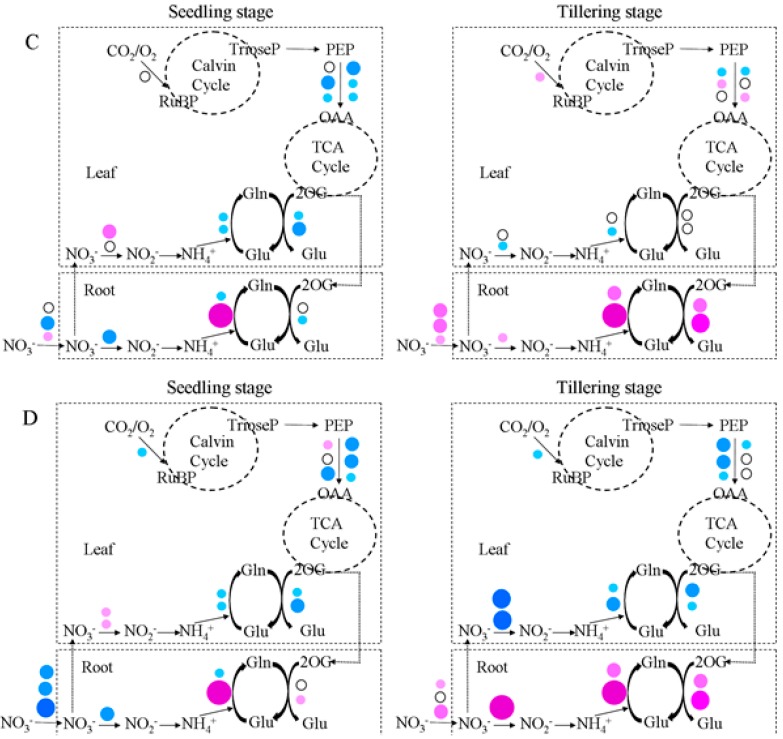
The fold change corresponds to the ratio of the gene expression level in the *GS2*-cosuppressed plants relative to the wild-type plants. (**A**) Diagrammatic representation of the key genes involved in the carbon and nitrogen metabolic pathway in rice plants. NRT, nitrate transporter; NR, nitrate reductase; GS, glutamine synthetase; GOGAT, glutamate synthase; RUBISCO, ribulose-1,5-bisphosphate carboxylase/oxygenase; PEPC, phosphoenolpyruvate carboxylase. Prominent changes in the gene expression level in the *GS2*-cosuppressed plants compared to wild-type plants at the seedling stage and tillering stage under N (**B**), G (**C**) and N + G (**D**) conditions. Red and blue dots indicate up- and down-regulated genes, respectively.

To determine the impact of the reduced *GS2* mRNA transcriptional level on the carbon-nitrogen metabolic genes’ expression patterns, we analyzed the expression level of genes encoding NRT (nitrate transporter), NR (nitrate reductase), GS (glutamine synthetase), GOGAT (glutamate synthase), RUBISCO (ribulose-1,5-bisphosphate carboxylase/oxygenase) and PEPC (phosphoenolpyruvate carboxylase) by q-RT PCR. Figure 6A displays these genes in the carbon and nitrogen metabolic pathway in rice plants. Supplementary Table S1 lists the fold change corresponding to the ratio of the gene expression level in *GS2*-cosuppressed plants relative to wild-type plants in the roots and leaves at both the seedling and tillering stage under N, G and N + G conditions. The results showed that the expression levels of most genes were changed in the *GS2*-cosuppressed plants. Compared to the wild-type plants, the significantly (*p* < 0.01) higher *GS2* expression levels (35.06–537.49-fold) were observed in the roots of *GS2*-cosuppressed plants at both the seedling stage and the tillering stage under N, G and N + G conditions (Supplementary Table S1). However, significantly (*p* < 0.05) lower *GS2* expression levels (0.43–0.79-fold) were observed in the leaves of *GS2*-cosuppressed plants at both the seedling stage and the tillering stage under the N, G and N + G conditions, except a higher *GS2* expression level (2.35-fold) was observed in the leaves at the seedling stage under the N condition (Supplementary Table S1). Furthermore, different gene expression patterns were exhibited in the *GS2*-cosuppressed plants between the seedling stage and tillering stage. Additionally, opposite gene expression patterns were shown between the root and leaf tissues, particularly at the tillering stage. For example, the expression levels of the *NRT1;1*, *NRT1;2*, *NRT2*, *NR2*, *GS1;2*, *Fd-GOGAT1* and *NADH-GOGAT1* genes were significantly (*p* < 0.05) increased in the roots, while the expression levels of the *NR1*, *NR2*, *GS1;1*, *GS2*, *Fd-GOGAT2*, *NADH-GOGAT2*, *RUBISCO*, *PEPC1* and *PEPC2* genes were significantly (*p* < 0.05) decreased in the leaves under the N condition (Figure 6).

## 3. Discussion

### 3.1. The Carbon-Nitrogen Metabolic Imbalance Is One of the Possible Reasons for the Chlorosis Phenotype in the GS2-Cosuppressed Plants

C and N are the essential elements for plant routine and fundamental growth and development. The optimal functioning and coordination of the C and N metabolism plays a very important role in plant growth and production [5,7,8,10]. Maintaining an appropriate balance or ratio of C and N nutrients is critical to sustain the optimal plant growth and development [5,8,10]. Because C and N resources change along with the environment, plants have developed a highly complex and sophisticated regulation system to adjust C and N uptake, transport and assimilation [10,37,38,39,40]. Schlüter *et al.* reported that the C, N and phosphorus balance played an important role in the stress adaptation of maize source leaf [41]. In the present study, our results showed an altered carbon and nitrogen metabolism in *GS2* transgenic plants. There were significant (*p* < 0.05) decreases of the carbon/nitrogen ratio in the stem tissue of *GS2*-cosuppressed plants. Furthermore, the opposite results between soluble protein and carbohydrate concentrations in the *GS2*-cosuppressed plants at both the seedling and the tillering stage indicated that the altered *GS2* expression level affected the carbon and nitrogen metabolic status. This type of carbon-nitrogen metabolic imbalance may be one of the reasons for the chlorosis phenotype of the *GS2*-cosuppressed plants. Early studies reported similar results that the C/N ratio, rather than the carbohydrate status alone, played the major role in the fresh weight, cotyledon size, chlorophyll and anthocyanin content in *Arabidopsis* [9]. Bao *et al.* reported that an imbalance of C-N metabolic status may cause the poor growth and yield in the *GS1;1*- and *GS1;2*-overexprssing transgenic rice [42]. Kusano *et al.* also reported that the rice *OsGS1;1* mutant showed an imbalance in tricarboxylic acid cycle metabolites, amino acids and sugars [43]. Additionally, variations in the leaf SPAD value, photosynthetic parameters, individual sugars, amino acids, organic acids and gene expression levels were observed in *GS2*-cosuppressed plants when compared to the wild-type plants in this study. Pérez-Delgado *et al.* also reported that the mutant plants lacking the plastidic isoform of Gln synthetase in *Lotus japonicas* accumulated high levels of several amino acids and organic acids, including intermediates of the Krebs cycle under photorespiratory active conditions [44].

### 3.2. The Incoordination between the Root and Leaf Tissues Is Another Possible Reason for the Chlorosis Phenotype in GS2-Cosuppressed Plants

In higher plants, a complex picture is emerging in which C/N signaling networks are subject to a “matrix effect” in which certain functions or interactions only occur under situations that are specific to a species, cell-type, developmental stage, metabolic status or environmental condition [8]. In addition to maintaining the carbon-nitrogen balance in the same organ and/or tissue, the tight coordination involves the sensing and signaling of the carbon/nitrogen balance between the source and sink tissues [5,8]. The NO_3_^−^ and/or NH_4_^+^ are usually taken up by the root system and transported to the leaves for the inorganic nitrogen assimilation, while carbon metabolism primarily occurs in the leaves to provide both the carbon skeletons and energies for nitrogen assimilation [5,8]. Therefore, plants must develop a mechanism to sense the status of the nitrogen in the root system and the surrounding soil environment and coordinate with the sensory machinery in the leaves, where the photosynthetic output will be determined [5,8]. In this study, compared to the wild-type plants, the total nitrogen content of *GS2*-cosuppressed plants showed no significant difference in the root, while significantly increased in the stem and decreased in the leaf. This suggested that *GS2*-cosuppressed plants had similar capacities for nitrogen uptake from the environment and nitrogen export from the root to the stem, and they had poor nitrogen transport ability from the stem to the leaf, which led to dramatic nitrogen accumulation in the stem. Furthermore, the expression pattern of the *GS2* gene and other key genes involved in carbon and nitrogen metabolism was inconsistent between the root and leaf in the *GS2*-cosuppressed plants. These results showed an incoordination between the root and leaf tissues in *GS2*-cosuppressed plants. We hypothesized that this may be another reason for the chlorosis phenotype of the *GS2*-cosuppressed plants.

### 3.3. The Impact of Gln on Plant Growth and Carbon-Nitrogen Metabolism

Plants can capture nitrogen in a variety of different chemical forms, ranging from inorganic forms, such as NO_3_^−^ and NH_4_^+^, to as many as 20 different amino acids [45,46,47,48,49,50,51]. The uptake rates for amino acids are considerably lower compared to inorganic forms of nitrogen [45,47,48,51]. In general, it appears that plants can use most of the amino acids as a source of nitrogen, which depress plant growth relative to mineral sources of nitrogen [45,52]. Only a minority of amino acids can occasionally increase plant growth [50,52] or at least match plant growth on mineral forms of nitrogen [53]. In our study, we analyzed the growth phenotype, carbon-nitrogen metabolic status and gene expression profile of *GS2*-cosuppressed rice and wild-type plants under three different nitrogen conditions (N, G and N + G). Results showed that both the *GS2*-cosuppressed rice and wild-type plants could grow under Gln as the nitrogen source. However, Gln depressed the plant growth, particularly the root length and root dry weight. When compared to the N condition, the leaf SPAD value, total carbon or nitrogen content increased, while the leaf stomatal conductance, intercellular CO_2_ concentration, transpiration rate and carbon/nitrogen ratio decreased under the G or N + G conditions. Additionally, Gln affected the gene expression patterns, particularly at the seedling stage. Similar results have been reported showing that, while plants generally have the capacity to take up many amino acids, these often negatively interfere with biomass production and even inhibit growth [54]. The model plant *Arabidopsis* has the capacity to acquire and utilize a number of amino acids for growth, including the amide glutamine (Gln) [48]. When grown with amino acids as the sole nitrogen source, *Arabidopsis thaliana* was only capable of achieving between 1% and 50% of its potential vegetative growth on an identical concentration of nitrate [48]. Results indicated that plants would be capable of growing when fed Gln, but Gln would contribute relatively little to plant growth compared to inorganic nitrogen sources of NO_3_^−^ and NH_4_^+^.

## 4. Experimental Section

### 4.1. Plant Materials and Growth Conditions

Seeds of the *GS2*-cosuppressed rice (the homozygous of T_2_ generation of transgenic Line 87 with 28% decreased GS activity in leaves was used in this study) and wild-type Zhonghua 11 (*Oryza sativa* ssp. *japonica*) were germinated and sowed in sand. Seedlings with two leaves were then transplanted hydroponically in a normal nutrient solution using 1.44 mM·NH_4_NO_3_ as a nitrogen source (N), a nutrient solution without NH_4_NO_3_, but using 2.0 mM glutamine (Gln) as a nitrogen source (G), and a nutrient solution using both 1.44 mM·NH_4_NO_3_ and 2.0 mM·Gln as nitrogen sources (N + G). The composition of normal nutrient solution was described in detail by Yoshida *et al.* [55]. At the seedling stage (one week after transplanting) and the tillering stage (five weeks after transplanting), the roots, stems and leaves were harvested for the phenotype evaluation, the leaf SPAD value and photosynthesis capacity determination, the carbon-nitrogen metabolism and gene expression analysis. All of the plants were grown in the pot farm at Huazhong Agricultural University, Wuhan, China. The nutrient solution was refreshed every 3 days, and the pH value was kept at 5.5.

### 4.2. Nitrogen Uptake Assay

Seeds of the *GS2*-cosuppressed rice (transgenic Line 88 was used in this study) and wild-type Zhonghua 11 (*Oryza sativa* ssp. *japonica*) germinated and were sowed in sand in the pot farm at Huazhong Agricultural University. Seedlings with two leaves were transplanted into the normal nutrient solution described by Yoshida *et al.* [55]. Plants were transferred into a new nutrient solution, in which the NH_4_NO_3_ was replaced by ^15^NH_4_^15^NO_3_, at the tillering stage (five weeks after transplanting). After 1 h, 3 h, 8 h, 1 day and 3 days, three biological replicated samples of roots, stems and uppermost leaves were harvested for the total N and ^15^N concentration determination. A C/N analyzer (Elementar, Vario MAX CN, Germany) and an isotope mass spectrometer (ANCA-MS, Europa Scientific, Crewe, UK) were used to measure the concentrations of total C/N and ^15^N, respectively, as described in detail previously [42].

### 4.3. Determination of the Leaf SPAD Value and Photosynthetic Parameters

As described in detail previously [42], every ten randomly-selected plants of the *GS2*-cosuppressed rice and wild-type Zhonghua 11 grown under the N, G and N + G conditions at the tillering stage (five weeks after transplanting) were used for the leaf SPAD value and photosynthetic capacity determination. The SPAD value and the photosynthetic rate, transpiration rate, intercellular CO_2_ concentration and stomatal conductance of upper, middle and bottom portion of the flag leaf in each plant were tested by a chlorophyll meter (SPAD-502) and a Li-6400XT portable photosynthesis system (Li-COR, Lincoln, NE, USA), respectively. The average mean was used in the further data analysis.

### 4.4. Determination of the Physiological Parameters

At the seedling stage (one week after transplanting) and the tillering stage (five weeks after transplanting), three biological replicated roots, stems and uppermost leaves of the *GS2*-cosuppressed rice and wild-type Zhonghua 11 grown under the N, G and N + G conditions were harvested for the concentrations of soluble proteins and carbohydrates analysis. All fresh samples were homogenized by grinding on ice with extraction buffer, which was described in detail previously [36,42]. After centrifuging (12,000× *g*, 20 min, 4 °C), the supernatant was used to analyze the concentration of soluble proteins by the Bradford method [56]. The pre-dried samples were boiled with boiling water to extract the soluble carbohydrates. Additionally, the concentration of soluble carbohydrates was measured colorimetrically by the anthrone method [57,58]. At the tillering stage (five weeks after transplanting), three biological replicated roots and uppermost leaves of *GS2*-cosuppressed rice and wild-type Zhonghua 11 grown under the N condition were harvested for the carbon-nitrogen metabolic profile analysis. Fifty milligrams of fresh sample were extracted with 1 mL extraction buffer (methanol/chloroform/water (3:1:1 *v*/*v*/*v*)) containing ten stable isotope compounds by a mixer mill at a frequency of 30 Hz for 3 min at 4 °C. The homogenates were then centrifuged at 15,000× *g* for 5 min at 4 °C. Four hundred microliters of supernatant were evaporated for further derivatization in a vacuum filled with 99.999% dry nitrogen. After evaporation, 20 μL methoxyamine hydrochloride were added to the sample for 30 h of derivatization at room temperature. Later, the sample was trimethylsilylated using 20 μL of MSTFA [*N*-Methyl-*N*-(trimethylsilyl)trifluoroacetamide] with 1% TMCS (Trimethylchlorosilane) at 37 °C with shaking for 1 h, and 20 μL *n*-heptane were added after silylation. The analysis of carbon and nitrogen metabolites by GC-TOF-MS and data analysis were performed as described by Kusano *et al.* [59,60] and Redestig *et al.* [61].

### 4.5. Gene Expression Analysis

At the seedling stage (one week after transplanting) and the tillering stage (five weeks after transplanting), three biological replicated roots and uppermost leaves of the *GS2*-cosuppressed rice and wild-type Zhonghua 11 grown under the N, G and N + G conditions were harvested for total RNA extraction (TRIzol reagent, Invitrogen, Darmstadt, Germany) and q-RT PCR analysis. The total RNA was treated by DNase I to remove the genomic DNA. The Superscript II reverse transcriptase (Invitrogen) was used to synthesize the first-strand cDNAs for q-RT PCR. The Primer Express Software (Foster City, CA, USA) and BLAST program in the database of the Institute for Genomic Research (TIGR, Available online: http://rice.plantbiology.msu.edu/) were used to design primers and check their specificity. Supplementary Table S2 lists the specific primers, both for tested genes and the rice *actin* gene (NM_197297). All of the experimental procedures were described in detail previously [36,42].

### 4.6. Statistical Analysis

The data were assessed with one-way ANOVA at *p* < 0.05 or *p* < 0.01 using SPSS 17.0 (SPSS Inc., Chicago, IL, USA).

## 5. Conclusions

**Figure 7:**
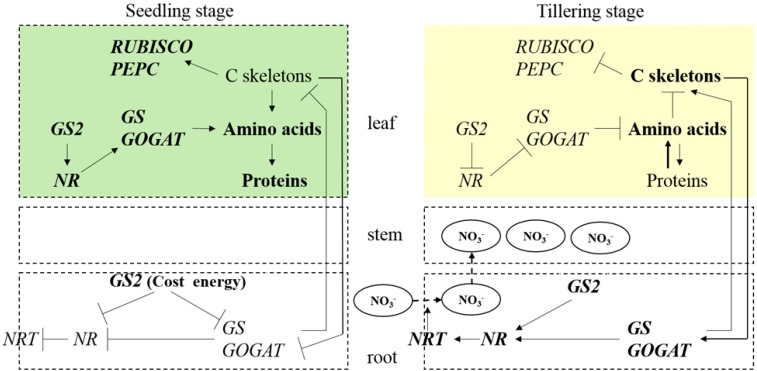
Summary of the differences between the seedling stage and the tillering stage in the *GS2* transgenic plant. The symbols ↑ and **⊺** indicate induction and repression, respectively.

In this study, we systematically analyzed the differences in the growth phenotype, carbon-nitrogen metabolic status and gene expression profile between *GS2*-cosuppressed rice and wild-type Zhonghua 11 at both the seedling stage and the tillering stage under three different nitrogen conditions (N, G and N + G). Results demonstrated the close relationships between the *GS2* mRNA levels and carbon-nitrogen metabolic status, as well as the plant growth phenotype. Figure 7 summarizes the differences between the seedling stage and the tillering stage in the *GS2* transgenic plants.

At the seedling stage, *GS2* was overexpressed under the control of the 35S promoter in the leaf tissue, which stimulated the expression levels of the *NR*, *GS* and *GOGAT* genes, resulting in an improvement in nitrogen reduction and assimilation for the increased production of amino acids and proteins. The content of carbohydrates decreased because of the large amount of carbon skeletons that were used to synthesize amino acids, and the expression levels of the *RUBISCO* and *PEPC* genes increased to produce more carbon skeletons. As fewer carbon skeletons could be transported into the root to participate in amino acid synthesis, the decreased *NRT*, *NR*, *GS* and *GOGAT* expression levels were displayed, which resulted in a decline in the nitrogen uptake, reduction and assimilation in the root. Additionally, *GS2* was excessively overexpressed (>35-fold) under the 35S promoter in the root, which could require a large amount of energy. This may be another factor causing the decreased nitrogen metabolic status in the roots of the *GS2* transgenic plants.

Although the *GS2* transgenic plants maintained a normal growth phenotype with green leaves at the seedling stage, the metabolic status was altered dramatically in both the root and leaf tissues because of the *GS2* overexpression. In this situation, plants must do something to maintain the metabolic status and complete the life cycle. From this point of view, the transgenic plants adjusted the *GS2* expression level in the leaves at the tillering stage. In the leaves, the *GS2* expression level decreased, which suppressed the *NR*, *GS* and *GOGAT* gene expression, resulted a decline in nitrogen reduction and assimilation, as well as decreased amino acid and protein production. However, the increased levels of free amino acids were observed in the metabolite profile analysis, which may have been caused by the protein degradation in the chlorotic leaves. In another case, the contents of the carbohydrates increased because few carbon skeletons were used to synthesize amino acids, and the expression levels of the *RUBISCO* and *PEPC* genes decreased to reduce the production of carbon skeletons. As more carbon skeletons were transported into the roots to participate in amino acids synthesis, increases in the *NRT*, *NR*, *GS* and *GOGAT* expression levels were observed, which resulted in an improvement in the uptake, reduction and assimilation of nitrogen in the roots. We concluded that the stable transcription level of *GS2* mRNA plays an important role in the normal development of rice and in the carbon-nitrogen metabolic balance. We hypothesized that the disrupted coordination of the carbon and nitrogen metabolic status and the excessively high expression level of *GS2* mRNA in the root were the possible reasons for the chlorosis phenotype in the *GS2*-cosuppressed plants.

## Supplementary Materials

Supplementary materials can be found at http://www.mdpi.com/1422-0067/16/06/12713/s1.
