# Supplementary material for: The Stable Level of Glutamine synthetase 2 Plays an Important Role in Rice Growth and in Carbon-Nitrogen Metabolic Balance
Source: Int J Mol Sci. 2015 Jun 4;16(6):12713–36. doi: 10.3390/ijms160612713 (PMC4490469; doi:10.3390/ijms160612713)
Supplement: Supplementary file 1 [file ijms-16-12713-s001.pdf]

# Supplementary Information

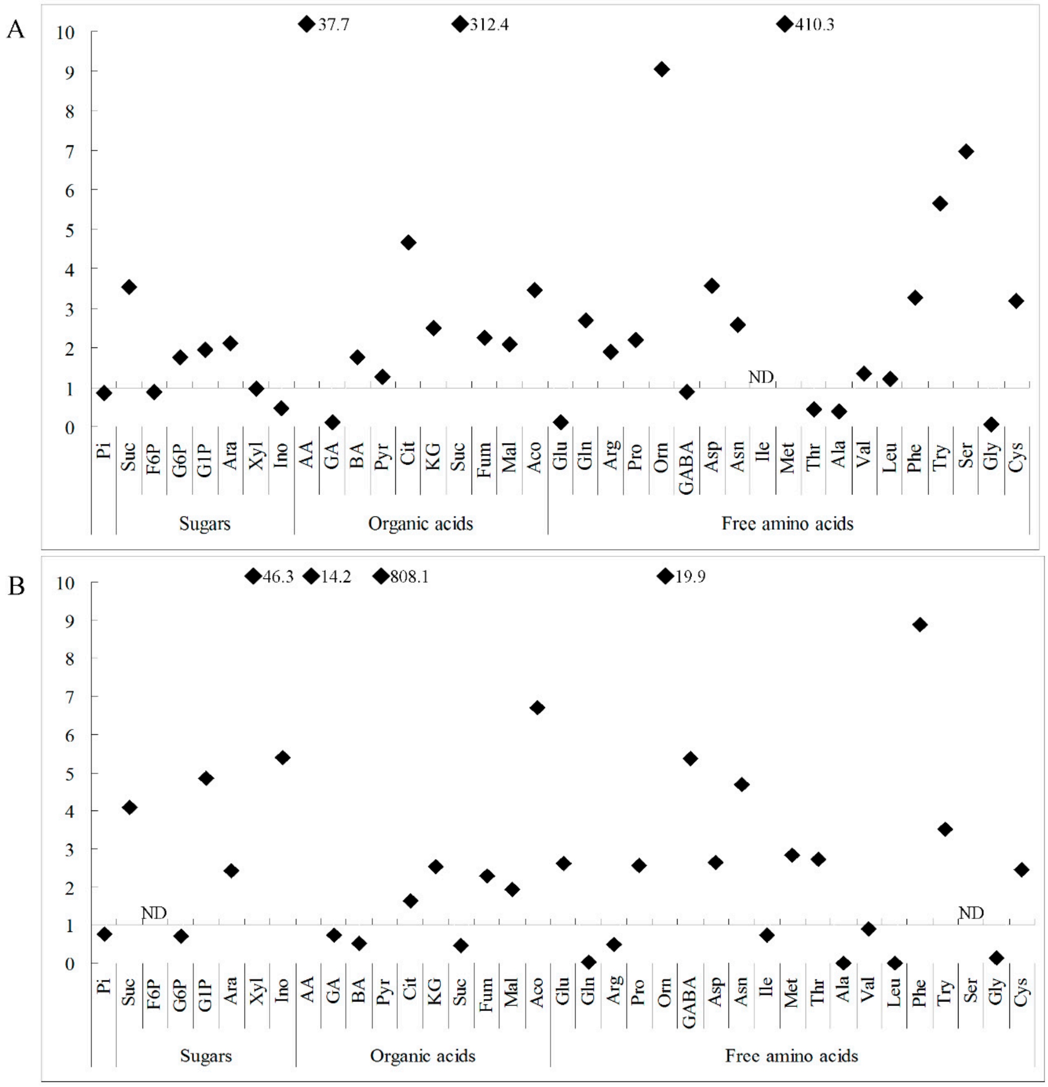


**Figure S1.** The fold change corresponds to the ratio of the concentration of individual metabolites involved in carbon and nitrogen metabolism in the *GS2*-cosuppressed plants relative to the wild-type plants for the leaves (**A**) and roots (**B**) at the tillering stage. Pi, phosphate; Suc (in the group of sugars), sucrose; Fru, fructose; F6P, frutose-6-P; G6P, glucose-6-P; G1P, glucose-1-P; Ara, arabinose; Xyl, xylitol; Ino, inositol; AA, ascorbic acid; GA, glutaric acid; BA, benzoic acid; Pyr, pyruvate; Cit, citrate; KG, ketoglutarate; Suc (in the group of organic acids), succinate; Fum, fumarate; Mal, malate; Aco, aconitase; Glu, glutamate; Gln, glutamine; Arg, arginine; Pro, proline; Orn, ornithine; GABA, aminobutyric; Asp, aspartate; Asn, asparagine; Ile, isoleucine; Met, methionine; Thr, threonine; Ala, alanine; Val, valine; Leu, leucine; Phe, phenylalanine; Try, tryptophan; Ser, serine; Gly, glycine; Cys, cysteine.

**Table S1.** The fold change corresponding to the ratio of the gene expression level in the *GS2*-cosuppressed plants relative to the wild-type plants for the roots and leaves at the seedling stage and the tillering stage under N ($NH_4NO_3$), G (Gln) and N + G ($NH_4NO_3$ + Gln) conditions.

| Genes | Seedling Stage | | | | | | Tillering Stage | | | | | |
|---|---|---|---|---|---|---|---|---|---|---|---|---|
| | $NH_4NO_3$ | | Gln | | $NH_4NO_3$ + Gln | | $NH_4NO_3$ | | Gln | | $NH_4NO_3$ + Gln | |
| | Root | Leaf | Root | Leaf | Root | Leaf | Root | Leaf | Root | Leaf | Root | Leaf |
| *NRT1;1* | 0.54 ** | 1.42 ** | 1.29 | 0.48 ** | 0.49 ** | 0.42 ** | 5.36 ** | 0.60 ** | 4.00 ** | 0.69 ** | 1.50 ** | 0.46 ** |
| *NRT1;2* | 1.23 | 0.17 ** | 0.45 ** | 1.60 ** | 0.39 ** | 0.06 ** | 2.09 ** | 1.55 ** | 4.83 ** | 1.17 | 1.94 | 1.02 |
| *NRT2* | 0.60 * | 0.23 ** | 1.82 ** | 0.93 | 0.13 ** | 0.21 ** | 2.87 ** | 0.68 * | 1.66 ** | 5.40 ** | 2.17 ** | 3.82 ** |
| *NR1* | 1.73 ** | 1.99 ** | 0.33 ** | 3.10 ** | 1.25 | 1.78 ** | 1.46 | 0.31 ** | 0.67 | 0.85 | 3.26 ** | 0.12 ** |
| *NR2* | 0.70 ** | 1.72 ** | 0.33 ** | 0.98 | 0.31 ** | 1.46 * | 1.56 ** | 0.53 ** | 1.99 * | 0.73 ** | 26.83 ** | 0.11 ** |
| *GS1;1* | 0.77 | 1.57 ** | 0.51 * | 0.79 ** | 0.84 * | 0.75 ** | 9.05 ** | 0.72 * | 1.48 ** | 1.17 | 2.06 ** | 0.66 ** |
| *GS1;2* | 0.82 ** | 1.97 ** | 0.68 ** | 0.68 * | 0.60 ** | 0.68 ** | 1.22 * | 0.99 | 3.24 ** | 1.39 ** | 3.15 ** | 0.86 |
| *GS1;3* | 1.97 ** | 0.48 ** | 0.49 ** | 4.47 ** | 1.73 ** | 0.21 ** | 2.16 ** | 0.59 * | 1.95 ** | 1.01 | 1.41 * | 7.19 ** |
| *GS2* | 35.06 ** | 2.35 ** | 65.44 ** | 0.79 * | 40.77 ** | 0.76 ** | 475.34 ** | 0.43 ** | 91.91 ** | 0.61 ** | 537.49 ** | 0.48 ** |
| *Fd-GOGAT1* | 0.75 ** | 1.35 ** | 0.72 | 0.63 ** | 1.13 | 0.48 ** | 10.97 ** | 0.64 ** | 4.68 ** | 1.29 | 2.33 ** | 0.62 ** |
| *Fd-GOGAT2* | 0.80 * | 1.45 ** | 0.86 | 0.67 * | 0.56 ** | 0.73 * | 6.74 ** | 0.42 ** | 1.94 ** | 1.03 | 9.95 ** | 0.33 ** |
| *NADH-GOGAT1* | 1.00 | 1.03 | 0.66 ** | 0.97 | 1.32 ** | 0.83 * | 5.77 ** | 1.63 ** | 6.23 ** | 0.64 ** | 5.24 ** | 0.99 |
| *NADH-GOGAT2* | 0.73 ** | 0.36 ** | 0.37 ** | 0.21 ** | 0.53 ** | 0.21 ** | 19.78 ** | 0.35 ** | 9.43 ** | 0.93 | 13.2 ** | 0.59 ** |
| *RUBISCO* | 0.43 ** | 1.89 ** | 1.67 ** | 0.80 | 1.30 * | 0.64 ** | 21.95 ** | 0.56 ** | 2.71 ** | 1.70 ** | 25.76 ** | 0.79 * |
| *PEPC1* | 2.16 ** | 1.38 ** | 2.10 * | 0.93 | 0.37 ** | 1.71 ** | 8.17 ** | 0.49 ** | 1.83 ** | 0.59 * | 46.93 ** | 0.35 ** |
| *PEPC2* | 0.71 * | 0.98 | 0.54 ** | 0.50 ** | 0.52 ** | 1.11 | 6.70 ** | 0.32 ** | 3.60 ** | 1.25 ** | 7.65 ** | 0.30 ** |
| *PEPC3* | 0.41 ** | 2.99 ** | 0.38 ** | 0.55 ** | 3.04 ** | 0.34 ** | 2.30 * | 1.41 ** | 1.86 ** | 0.99 | 1.53 * | 0.70 ** |
| *PEPC4* | 0.60 ** | 1.23 | 0.89 | 0.48 ** | 2.67 ** | 0.36 ** | 6.70 ** | 1.27 | 3.84 ** | 0.71 * | 1.60 ** | 0.75 ** |
| *PEPC6* | 0.72 * | 1.60 * | 2.29 ** | 0.71 ** | 1.62 ** | 0.48 ** | 7.88 ** | 0.96 | 1.23 ** | 1.03 | 19.86 ** | 0.92 |
| *PEPC7* | 0.53 ** | 1.24 * | 0.88 * | 0.53 ** | 3.01 ** | 0.62 ** | 7.21 ** | 0.85 | 1.80 ** | 1.25 * | 3.03 ** | 0.85 |

Values are mean from three independent experiments using three randomly mixed plant materials. *, ** Significant differences at the level of $p = 0.05$ and $p = 0.01$, respectively. NRT: nitrate transporter; NR: nitrate reductase; GS: glutamine synthetase; GOGAT: glutamate synthase; RUBISCO: Ribulose-1,5-bisphosphate carboxylase/oxygenase; PEPC: phosphoenolpyruvate carboxylase.

**Table S2.** Primer sequences of the key genes involved in the carbon and nitrogen metabolism used in qRT-PCR.

| Gene Name | cDNA Accession No. | Primer Sequence (5′–3′) |
|---|---|---|
| *NRT1;1* | AK066920 | F: CCTCGCAAGTGACCCTTGAAT<br>R: CGATGGCTAATGAGGAACCCTT |
| *NRT1;2* | AK101480 | F: GAACATGCGGATCATGTCGTT<br>R: CGATCACGGAGCTGTACATGAG |
| *NRT2* | AK109733 | F: TTCGCGAACCCGCATATGA<br>R: GTTGAGGTTGTCGCGGATGAT |
| *NR1* | AK102178 | F: ACTACCATTACCGCGACAACC<br>R: CTCGTTTATCATGTACTCCGGC |
| *NR2* | AK121810 | F: AGCTGAACGTGAACTCGGTGA<br>R: AGGCGTATCCCTTCATGGTGT |
| *GS1;1* | AK109397 | F: GAGTCGTCGTCTCATTTGACCC<br>R: GTAGCCACCATCGTTCCTCATC |
| *GS1;2* | AK243037 | F: TTTTCAAGGACCCGTTCAGGA<br>R: CGGCACTGTGCCTCTTGTTAGT |
| *GS1;3* | AK099290 | F: TCAAGCCATCTTCAGAGACCCA<br>R: TACCGGTTGTTCGTCGGAATC |
| *GS2* | AK063706 | F: AGGATCGGACAAATCGTTTGG<br>R: GCATGACCTCTCCATTTGTTCC |
| *Fd-GOGAT1* | AK102025 | F: AAATGCCTCTTTGCAAGGCC<br>R: GACTGTGAG CCCCATCCAAATA |
| *Fd-GOGAT2* | AK068130 | F: CCGATGCGATTGAGAATGAGA<br>R: CTTCTTGGCAATGACACCTGC |
| *NADH-GOGAT1* | AK105755 | F: TGCTTGAGAGAATGGCGCA<br>R: AACCCAGCATCCTTTGTCACC |
| *NADH-GOGAT2* | AK070485 | F: GGTTGTCATTGGTGGTGGAGA<br>R: TGGTGGCTCTGGCAAAAGTT |
| *RUBISCO* | AK243615 | F: AGGCTTCAAATTGCCGTTGA<br>R: TCTAGGCCATCCAGTTCCTCCT |
| *PEPC1* | AK100688 | F: ACATTCCGTGTTGCTGCAGAG<br>R: TGCAACAGTTCAACCGCTAGG |
| *PEPC2* | AK066635 | F: CAGAAGCACGCAAGCATTAGG<br>R: CGCGAGAATCTCTCTCTGAAGG |
| *PEPC3* | AK101274 | F: ACCGGTCCATTGTCTTCCAAG<br>R: CGTTTTGATGGCCTACTTCCAA |
| *PEPC4* | AK065425 | F: TGGATGAGATGGCTGTTGTGG<br>R: TTCTGTCTCAGGTGTTGCCGA |
| *PEPC6* | AK073703 | F: ATGTCTGCCAGGCTTACACGAT<br>R: CGGCTTAGACCAGTCCATGATC |
| *PEPC7* | AK242583 | F: GAGTATTTCCGCCTTGCAACAC<br>R: ACGGAGTGATTCAATGCCTCC |
| *ACTIN* | AK070531 | F: GACAATGGAACCGGAATGGTC<br>R: CCCAACCATAACGCCTGTATGT |
